# PKA modulation of Rac in neuronal cells

**DOI:** 10.3389/fncel.2014.00321

**Published:** 2014-10-14

**Authors:** Akihiro Goto, Yuji Kamioka, Michiyuki Matsuda

**Affiliations:** Department of Pathology and Biology of Diseases, Graduate School of Medicine, Kyoto UniversityKyoto, Japan

**Keywords:** PKA, Rac, guanine nucleotide exchange factor, migration

The Rho-family GTPase, Rac, is a molecular switch that controls actin dynamics and thereby the morphology, migration, and cytokinesis of most, if not all, cell types (Heasman and Ridley, 2008). Neuronal cells are not an exception, although Rac-regulated replication and migration are limited mostly to the embryonic stages (Luo, 2000; Tashiro and Yuste, 2004; Fuchs et al., 2009; Govek et al., 2011). Recently, it has also been shown that Rac is required for proliferative production and retention of new neurons generated during learning, indicating that Rac also regulates higher brain function (Haditsch et al., 2013). This small molecular switch is able to bring about so many different outcomes because it is embedded in many circuits, each of which comprises of a number of signaling molecules. Therefore, the function and regulation of Rac are inevitably cell context-dependent.

Protein kinase A (PKA), a canonical signal transducer of cAMP, plays pivotal roles in neuronal outgrowth, survival and regeneration (Qiu et al., 2002) and in axonal guidance (Song et al., 1998; Tojima et al., 2011). PKA activity is also required for the migration of enteric neural crest-derived cells during development of the enteric nervous system (Barlow et al., 2003; Asai et al., 2006). These observations suggest that PKA may regulate Rac to induce s a number of morphological changes. Here, we focus on the regulation of Rac by PKA in neuronal cells.

The direct upstream input to Rac comes from guanine nucleotide exchange factors (GEFs) and GTPase-activating proteins (Figure 1A). Among Ras-superfamily small GTPases, Rac, and Cdc42, a close relative of Rac1, are unique in that they are activated by two structurally unrelated families of GEFs, the classical Dbl homology-pleckstrin homology domain-containing GEFs (Cook et al., 2014) and the DOCK180-related atypical GEFs (Laurin and Cote, 2014). Among the more than 60 Dbl-family GEFs, Tiam1, STEF/Tiam2, P-Rex1, and Vav3 have been shown to regulate the migration of neuronal progenitor cells and neurite extension of neurons (Govek et al., 2011). On the other hand, studies with knockout mice or gene silencing of DOCK-family genes have revealed that DOCK3/MOCA deficiency leads to axonal degeneration and sensorimotor impairments (Chen et al., 2009), that DOCK6 is required for axon extension in dorsal root ganglion neurons (Miyamoto et al., 2013), and that DOCK7 regulates the interkinetic nuclear migration of radial glial progenitor cells (Yang et al., 2012).

**Figure 1:**
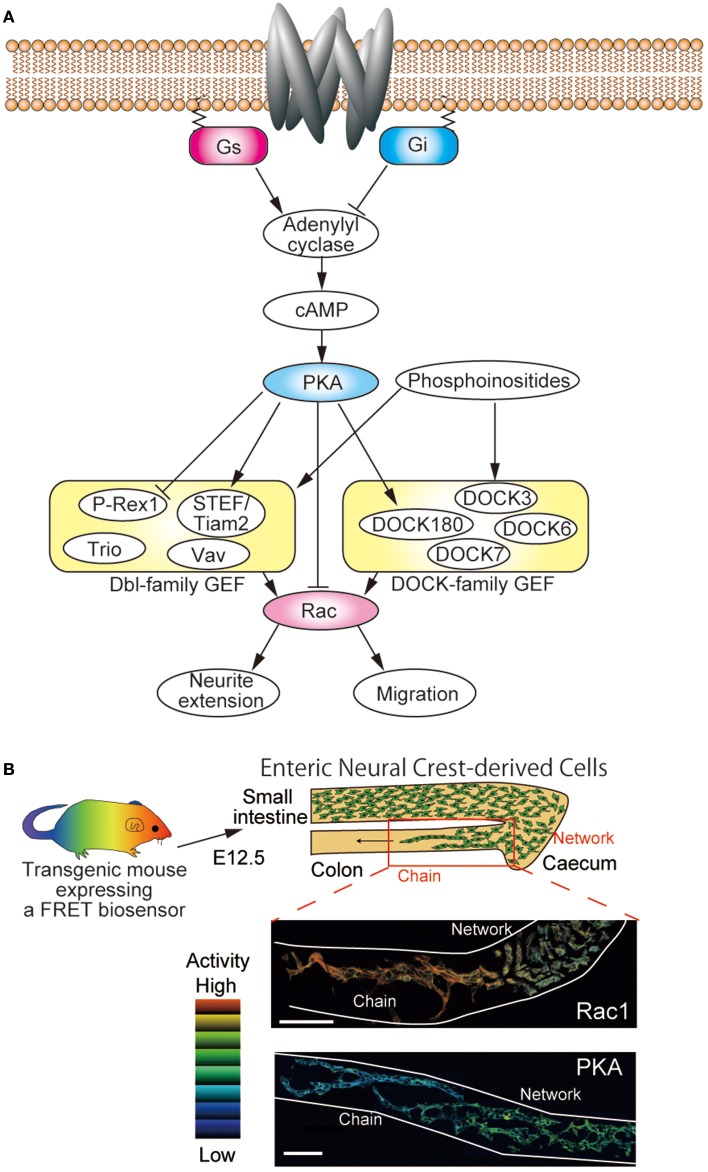
**Regulation of Rac by PKA in neuronal cells. (A)** Schematic view of the regulatory network of Rac in neuronal cells. Neuronal cells express both Dbl-family and DOCK-family GEFs. Among them, P-Rex1, STEF/Tiam2, and DOCK180 have been shown to be phosphorylated by PKA. Rac activity is required for neurite extension, migration, and so on. **(B)** Transgenic mice expressing FRET biosensors can be used to examine the *in vivo* activities of Rac1 and PKA. Embryonic intestines of E12.5 derived from transgenic mice expressing a FRET biosensor for either Rac1 or PKA were observed under two-photon microscopes. FRET images were prepared to show the activity of Rac1 and PKA. Enteric neural crest cells (ENCCs) migrate from the stomach to the colon during development. The ENCCs that migrate rapidly in chains exhibit higher Rac1 activity and lower PKA activity than the ENCCs that form a neural network.

At one level above GEF are various serine/threonine kinases, which phosphorylate GEFs and thereby activate or inactivate them. PKA is a good candidate for such a serine/threonine kinase; however, there have been no direct demonstrations of PKA regulation of GEFs for Rac, except for a few examples, in neuronal cells. In PC12D cells, PKA phosphorylates and activates a Rac GEF, STEF/Tiam2, and induces neurite extension (Goto et al., 2011). On the other hand, phosphorylation of P-Rex1, another GEF, has been shown to suppress GEF activity in HEK293T cells (Mayeenuddin and Garrison, 2006; Barber et al., 2012). Although knockout mice of P-Rex1 exhibit cerebellar dysfunction (Donald et al., 2008), it is unknown whether the PKA-P-Rex1 axis operates in neuronal cells. Less is known about the direct link between PKA and DOCK-family GEFs. Only recently it has been shown that, in glioblastoma cells, PKA phosphorylates and stimulates DOCK180 and thereby promotes growth and invasion (Feng et al., 2014).

Additional complexity comes from the regulation by phosphoinositides and phosphatidic acid. Although the Dbl-family GEFs and the DOCK-family GEFs are structurally unrelated, both GEFs are regulated by phosphoinositides or phosphatidic acid, which are bound to the PH domain in the Dbl-family GEFs or the DHR-2 domain in the DOCK-family GEFs (Cook et al., 2014; Laurin and Cote, 2014). Considering the pleiotropic effects of PKA, it is likely that PKA regulates Rac by controlling the levels of phosphoinositides and phosphatidic acid. For example, PKA has been shown to activate phosphatidylinositol 3-kinase (PI3K) via p85 subunit phosphorylation (Cosentino et al., 2006). In another report, however, PKA was shown to inhibit PI3K via p110 subunit phosphorylation (Perino et al., 2011). Therefore, the effect of PKA on PI3K is cell context-dependent. The development of probes for phosphoinositides and phosphatidic acid will help to resolve this issue (Nishioka et al., 2010). Finally, PKA can directly inhibit Rac1 by phosphorylating at Ser 71 during bacterial infection (Brandt et al., 2009).

Furthermore, Bachmann et al. have reported a direct binding of Rac1 with PKA by GFP complementation assay (Bachmann et al., 2013). The PKA-Rac1 axis functions to activate ERK MAP kinase and thereby regulates cell proliferation. It should be studied further whether the PKA-Rac1 axis functions also in neuronal cells.

Due to the complexity and cell context-dependency of the regulatory networks where PKA and Rac are embedded, the role played by PKA in the regulation of Rac and the resulting morphological changes are almost entirely unpredictable *in vivo*. Thus, the activities of PKA and Rac should be examined together with biological outputs such as migration and neurite extension. To accomplish such comprehensive investigation, transgenic mice expressing biosensors based on the principle of Förster resonance energy transfer (FRET) were developed recently (Kamioka et al., 2012; Johnsson et al., 2014). By observing the embryonic intestines of transgenic mice expressing FRET biosensors for PKA and Rac1, clear reciprocal activation of PKA and Rac1 has been demonstrated in migrating enteric neural crest-derived cells (Figure 1B) (Goto et al., 2013). Currently, simultaneous observation of two FRET biosensors is a difficult task, but recent advent of bright infrared fluorescence will open a way to use two different FRET biosensors to visualize the interplay by two different signaling molecules in a single cell.

Further insight into the role played by PKA and Rac1 will be obtained by modulating the activity of Rac1 or PKA. Inhibitors against Rac1 and PKA are available; however, there is a critical problem for *in vivo* or *ex vivo* experiment. By the conventional bath application of reagents, we cannot tell whether the reagent affected the neuronal cells directly or indirectly via surrounding cells. Application of caged cAMP analogs or optogenetic tools such as light-activatable Rac1 (Wu et al., 2009) to the FRET biosensor-expressing mice will overcome this problem and help us to untangle the complex signaling networks that regulate the morphological changes of neuronal cells.

## Conflict of interest statement

The authors declare that the research was conducted in the absence of any commercial or financial relationships that could be construed as a potential conflict of interest.
